# Analysis of factors influencing college students’ food waste behavior and evaluation of labor education intervention

**DOI:** 10.3389/fpubh.2024.1372430

**Published:** 2024-05-15

**Authors:** Dandan Wang, Kaiyue Zhang, Xin Lv, Liang Xue, Zhenyu Yang, Peng Li

**Affiliations:** ^1^School of Public Health, Xuzhou Medical University, Xuzhou, Jiangsu, China; ^2^Center for Medical Statistics and Data Analysis, Xuzhou Medical University, Xuzhou, Jiangsu, China; ^3^Key Laboratory of Human Genetics and Environmental Medicine, Xuzhou Medical University, Xuzhou, Jiangsu, China; ^4^Xuzhou Engineering Research Innovation Center of Biological Data Mining and Healthcare Transformation, Xuzhou Medical University, Xuzhou, Jiangsu, China; ^5^Jiangsu Engineering Research Center of Biological Data Mining and Healthcare Transformation, Xuzhou Medical University, Xuzhou, Jiangsu, China; ^6^Yangzhou Center for Disease Control and Prevention, Yangzhou, Jiangsu, China; ^7^School of Basic Medical Sciences, Harbin Medical University, Harbin, Heilongjiang, China; ^8^Department of Public Health and Preventive Medicine, Changzhi Medical College, Changzhi, Shanxi, China

**Keywords:** food waste, labor education, college students, survey, interventions

## Abstract

**Background:**

Food waste remains a major problem for the world and food security. Despite the fact that consumers are significant producers of food waste, little research attention has been paid to college students. The present study aimed to assess food waste and the influence factors among college students. Additionally, the goal was to improve college students’ food waste attitudes and behaviors through labor education.

**Methods:**

Through an online questionnaire survey, 407 college students from three universities were asked about food waste; 27 students of them were randomly selected as the research object, and labor practice was carried out in groups in the student cafeteria. Mann–Whitney U test was performed to show food waste behavior of college students and logistical regression analysis was used to analyze the factors affecting food waste behavior.

**Results:**

The results indicated that the food waste is more serious among college students in East China, senior or female students, BMI plays a positive role in food waste among college students, while monthly consumption and peers waste play a negative role in food waste. After participating in the labor education, the students’ views and practices toward their peer’s food waste have improved.

**Conclusion:**

The implementation of labor education in college canteens is conducive to the establishment of a correct outlook on labor as well as saving consciousness among college students, and to the formation of a social consciousness of saving.

## 1 Introduction

There’s an old Chinese saying, “Food is the God of the people.” Food is a basic necessity for everyone as it provides them with survival and health. Meanwhile, it has an irreplaceable basic position in the development of the national economy. Food production and supply, therefore, is a major world-class topic. However, due to wars, diseases, extreme weather and other factors, access to food is still partially or significantly limited in many regions of the world, which threatens the well-being of societies. It was estimated in 2020 that from 720 to 811 million people worldwide experienced hunger, which is as much as 161 million people more than the year before considering the upper end of this range (1). However, China is a large agricultural country with 1.4 billion people and 800 million farmers, the issue of food security is particularly important.

On the other hand, however, food waste is a global problem. According to (2), “food waste refers to food fit for human consumption that is discarded, whether or not it is stored beyond its expiry data or left to spoil. This is often due to food going bad, but can also be due to other reasons such as oversupply in markets of individual consumer shopping/eating habits.” According to the 2011 report of the Food and Agriculture Organization of the United Nations (3), global food waste generation is estimated at roughly 1.3 billion tons of still edible food is wasted every year, one third of food suitable for eating by humans was wasted every year in the first decade of the 21st century, with a 44% predicted increase between 2005 and 2025 (4). In China, with the improvement of the living standard of the population and the rise in consumption capacity, the phenomenon of food waste is also becoming increasingly serious. According to the report made by the Institute of Science and Technology Strategic Consulting of the Chinese Academy of Sciences in 2020, China’s annual food waste is approximately 67.5 million kg (5).

Food waste is said to occur at all stages of the food chain, 28% of waste occurs at the consumer level (6). Despite the fact that consumers are significant producers of food waste, little research attention has been paid to college students. China has a large number of college students, and some researchers have found that up to 74% of college students have food waste behavior, *per capita* food leftover weight reached 61.03 g/meal, the average food waste rate per person per meal is 12.13% (7).

Labor education in the new era, as an important way to implement the fundamental task of establishing moral education, is an important motivation to inspire college students to aspire to success. Therefore, colleges should increase student’ social practice experience, cultivate students’ ideology of respect for labor, organize students to go out of the classroom and carry out a wide range of practical experience activities.

If the citizens of this country do not pay enough attention to food waste, it is likely that food waste will grow gradually as people’s living standards are improving year after year. In addition to the environmental and pollution problems caused by food waste, it may also lead to a series of social problems. Therefore, this study aims to study the current situation of food waste behavior of college students and the analysis of influencing factors, so as to reduce food waste behavior of college students by participating in the practice of labor education and improve their consumption concept, which is contribute to the development of China’s society, economy, culture and ecology.

## 2 Materials and methods

### 2.1 Methodical approach

An online survey was conducted to collect sample for this research work to obtain the current situation of food waste behavior among college students, which was showed in Table 1. The idea of food waste was clearly explained to the respondents at initial stage of the survey, so they clearly understand what was and was not. The questions in the questionnaire included socio-demographic aspects, aspects of family trait, dining characteristics, as well as environmental characteristics. The questionnaire was adopted from an earlier research paper (8), with a slight modification based on this research requirement. According to the latest statistics of the Ministry of Education of the People’s Republic of China on number of students of formal education by type and level, there are currently more than 19.65 million undergraduate students enrolled in colleges, including 1.29 million medical students. The participants were randomly selected from students at Changzhi Medical College in North China, Harbin Medical University in Northeast China, and Xuzhou Medical University in East China. The online survey was conducted from 9 to 16 October 2023. In all, 407 students took part in the survey from different universities in China, accounting for 0.03% of all medical students.

**Table 1:** Variable assignment of factors influencing college students’ food waste.

Type	Name	Variable assignment
Dependent variable 1	Level of food waste per meal 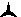	1 = Yes; 2 = No
Dependent variable 2	Frequency of food waste per week 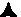	1 = Yes; 2 = No
Personality characteristics	Region of the University	1 = North China; 2 = Northeast; 3 = East China
	Gender	1 = Male; 2 = Female
	BMI^#^	1 = Underweight; 2 = Normal weight;3 = Overweight; 4 = Obese
	Grade	1 = First year; 2 = Second year; 3 = Third year or more
	Scholarship	1 = Yes; 2 = No
	Hunger experience	1 = Yes; 2 = No
	Agricultural labor experience	1 = Yes; 2 = No
	Average monthly consumption (yuan)	1 = Less than 800; 2 = 800–1,500; 3 = 1,500-2,000; 4 = More than 2,000
	Attitude toward “Clean your plate campaign”	1 = Strongly disapprove, waste of time; 2 = Disagree, do not think it’s very effective; 3 = It does not matter, follow the crowd; 4 = Strongly support it and think it makes sense
Family trait	Household registration	1 = Urban; 2 = Rural
	An only child	1 = Yes; 2 = No
	Parental accompaniment	1 = Yes; 2 = No
	Family farming	1 = Yes; 2 = No
	Parental education	1 = Junior high school and below; 2 = Senior high school; 3 = Specialty; 4 = Undergraduate college; 5 = Postgraduate
Dining characteristics	Dining place	1 = Cafeteria; 2 = Dormitory; 3 = Dining out
	Number of people eating together	1 = Dining alone; 2 = 1 person sharing a meal; 3 = Shared meals for more than 2 people
	Peers waste	1 = Never; 2 = Once in a while; 3 = Often; 4 = Always
Environmental characteristics	Cafeteria vegetable quantity	1 = Very dissatisfied; 2 = Unsatisfactory; 3 = General; 4 = Relatively satisfied; 5 = Very satisfied
	Cafeteria meal quantity	1 = Very dissatisfied; 2 = Unsatisfactory; 3 = General; 4 = Relatively satisfied; 5 = Very satisfied

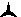
Level of food waste per meal. Yes = Waste a little or more per meal; No = Almost no waste per meal.

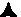
Frequency of food waste per week. Yes = Waste food more than 1 time per week; No = Waste food less than 1 time per week.

^#^BMI, body mass index. According to the Chinese Public Health Standards, as follows: underweight, BMI < 18.5 kg/m^2^; normal weight, 18.5 ≤ BMI < 24.0 kg/m^2^; overweight, 24 ≤ BMI < 28.0 kg/m^2^; and obese, BMI ≥ 28.0 kg/m^2^.

### 2.2 Model logit

While analyzing the influence of food waste in different college students (the dependent variable), a logit model (a qualitative model) was used, in which the dependent variable (Y) was “Level of food waste per meal” or “Frequency of food waste per week.” Variable Y in the logit model took the following values:

Y_1_ = 1—Food waste per meal.

Y_1_ = 0—No food waste per meal.

Y_2_ = 1—Weekly food waste.

Y_2_ = 0—No weekly food waste.

The results of the logit model estimation were interpreted based on: (a) the log odds ratio (LOR), (b) the odds ratio (OR) for each independent variable, through which the change in the odds of occurrence of the selected value is expressed (*Y* = 1) when the independent variable grows by 1 unit (*ceteris paribus*).

The quality of matching the model of the variable “Food waste” was evaluated on the basis of the determination coefficient (*R*^2^). The values of this coefficient fall within the range (0–1), and the higher the value, the better the matching of the model.

### 2.3 Labor education implementation

Using whole cluster random sampling, it is due to resource limitations, one natural class (27 students) of preventive medicine was identified as the research subjects to participate in this labor education activity. The subjects were randomly divided into three groups, namely, plate recycling group, table hygiene group and serving free soup group, and the understanding and requirements were unified before the activity. Among them, the plate recycling group was mainly responsible for dumping college students’ leftovers into a special food waste bin and classifying other garbage, the table hygiene group was a mobile operation mainly responsible for cleaning up the desktop hygiene after college students’ meals and the free soup group was mainly responsible for serving free soup to students in need. The students who participated in the labor education activity wore uniforms, hats, masks and gloves, and the work was stationed in the student cafeteria and carried out labor experience in their respective positions during the peak eating hours (Lunch: 11:30 ~ 12:30; Diner: 17:30 ~ 18:30) for a period of 3 days.

In order to further investigate the effectiveness of the labor education implementation, we repeated the survey on food waste behaviors with the 27 students mentioned above, with questions about changes in awareness of cafeteria waste, personal food waste behaviors, and attitudes toward food waste, and returned the online questionnaire within 1 day after the labor education implementation was completed.

### 2.4 Statistic methods applied

SPSS 19.0 statistical software was used to rationalize the differences in food waste behavior. Qualitative data were expressed as composition ratio (%). Comparison of differences in the current status of food waste among college students and the effectiveness of the implementation of labor education were tested using the nonparametric rank sum test (Mann–Whitney U test, $u=\frac{U-n_{1}n_{2}/2}{\sqrt{\frac{n_{1}n_{2}\left(N+1\right)}{12}\left(1-\frac{\sum \left({t_{j}}^{3}-t_{j}\right)}{N^{3}-N}\right)}}$). Logistics multivariate regression analysis was used to analyze the factors affecting food waste behavior of college students, which were used to analyze the odds ratio (OR) and 95% confidence intervals (CIs), and differences were considered statistically significant at *p* < 0.05.

## 3 Results

### 3.1 Characteristics of respondents

In this survey, 407 valid questionnaires were collected, balanced among students from three districts. Students between the ages of 18 and 25 participated in the study. More than half of the students who participated in the survey were freshmen, meaning that there was a higher level of participation from underclassmen. A larger percentage of respondents were female. Of the students who participated in the survey, 67.3% of them were normal weight, 11.1% were underweight, and the remaining 21.6% were overweight or obese (Table 2).

**Table 2:** Summary of the socio-demographic data about the participating students (China, 2023, *n* = 407).

Variable	Description	Frequency (%, *n*)
Region of the University	North China	29.0 (118)
	Northeast	35.1 (143)
	East China	35.9 (146)
Age (years)	18-	82.6 (336)
	20-	14.5 (59)
	22-	2.9 (12)
Grade	First year	52.3 (213)
	Second year	30.2 (123)
	Third year or more	17.4 (71)
Gender	Male	34.9 (142)
	Female	65.1 (265)
BMI (kg/m^2^)^#^	Underweight	11.1 (45)
	Normal weight	67.3 (274)
	Overweight	13.5 (55)
	Obese	8.1 (33)

^#^BMI, body mass index. According to the Chinese Public Health Standards, as follows: underweight, BMI < 18.5 kg/m^2^; normal weight, 18.5 ≤ BMI < 24.0 kg/m^2^; overweight, 24 ≤ BMI < 28.0 kg/m^2^; and obese, BMI ≥ 28.0 kg/m^2^. The results were shown as frequency.

### 3.2 The current situation of food waste among college students

The composition ratios of food waste per meal or the frequency of food waste per week varied among students in different schools. About half of the students in the three regions wasted hardly any food per meal and wasted food less than once a week. In North China, Northeast China and East China, the proportion of students who wasted less than 10% of food per meal was 94.1, 93, and 79.5%, respectively (Table 3). The proportion of food wasted less than 3 times per week was 96.6, 93.7, and 89.8%, respectively (Table 3). It can be seen that food waste among college students in East China is more serious than in the previous two regions.

**Table 3:** The current situation of food waste among college students (China, 2023, *n* = 407).

			Level of food waste per meal					Frequency of food waste per week			
		*N*	Almost no waste *n* (%)	Waste a little (<10%) *n* (%)	Less waste (10–30%) *n* (%)	Comparatively large amount of waste (30–50%) *n* (%)	Large amount of waste (>50%) *n* (%)	0–1 time per week *n* (%)	2–3 times per week *n* (%)	4–5 times per week *n* (%)	More than 5 times per week *n* (%)
		407	218 (53.6)	142 (34.9)	42 (10.3)	4 (1.0)	1 (0.2)	267 (65.6)	112 (27.5)	16 (3.9)	12 (3.0)
Region of the University	North China	118	71 (60.2)	40 (33.9)	5 (4.2)	1 (0.8)	1 (0.8)	87 (73.7)	27 (22.9)	1 (0.8)	3 (2.5)
	Northeast	143	78 (54.5)	55 (38.5)	10 (7.0)	0 (0)	0 (0)	98 (68.5)	36 (25.2)	4 (2.8)	5 (3.5)
	East China	146	69 (47.3)	47 (32.2)	27 (18.5)	3 (2.1)	0 (0)	82 (56.2)	49 (33.6)	11 (7.5)	4 (2.7)
	*χ* ^2^		119.96					10.41			
	*P*		<0.001^*^					0.005^*^			
Grade	First year	213	124 (58.2)	79 (37.1)	10 (4.7)	0 (0)	0 (0)	148 (69.5)	58 (27.2)	4 (1.9)	3 (1.4)
	Second year	123	56 (45.5)	44 (35.8)	19 (15.4)	3 (2.4)	1 (0.8)	79 (64.2)	28 (22.8)	8 (6.5)	8 (6.5)
	Third year or more	71	38 (53.5)	19 (26.8)	13 (18.3)	1 (1.4)	0 (0)	40 (56.3)	26 (36.6)	4 (5.6)	1 (1.4)
	*χ* ^2^		155.79					5.023			
	*P*		<0.001^*^					0.081			
Gender	Male	142	102 (71.8)	35 (24.6)	5 (3.5)	0 (0)	0 (0)	118 (83.1)	20 (14.1)	3 (2.1)	1 (0.7)
	Female	265	116 (43.8)	107 (40.4)	37 (14.0)	4 (1.5)	1 (0.4)	149 (56.2)	92 (34.7)	13 (4.9)	11 (4.2)
	*Z*		−14.90					−5.43			
	*P*		<0.001^*^					<0.001^*^			

The results were shown as frequency. Statistical analysis was conducted using the Mann–Whitney U test. ^*^p < 0.05 compared to the control group.

Food waste varies among college students in different grades. 50% of students do not waste any of their meals. As many as 95.3% of first year students waste less than 10%. 20% of second year and above students waste 10% or more per meal (Table 3). The level of food waste per meal and the frequency of food waste per week varied among college students of different genders. The majority of female students (56.2%) wasted different levels of food per meal, and 43.8% wasted food more than twice a week. Most male students (71.8%) wasted little at each meal, and 83.1% of male students wasted food less than once a week (Table 3).

In summary, food waste is more serious among college students in East China, senior or female student.

### 3.3 Analysis of the influencing factors of college students’ food waste

The results of the multilevel analysis of the factors affecting food waste among college students are shown in Table 4. The null model (referent) with no exploratory variable revealed a significant variation in food waste between college students in the multilevel linear regression models. The logistic model assessed the relationship between personality characteristics, family trait, dining characteristics and environmental characteristics with food waste. The results showed that students with higher levels of BMI were less likely to have food waste [OR (95% *CI*), 0.680 (0.497, 0.930), *p* = 0.016], students with higher levels of monthly consumption were more likely to have food waste [OR (95% *CI*), 1.438 (1.050, 1.968), *p* = 0.024], and peer waste exacerbated the waste of students who dined with them, either per meal [OR (95% *CI*), 1.924 (1.224, 3.022), *p* = 0.005] or weekly [OR (95% *CI*), 1.617 (1.021, 2.561), *p* = 0.040] of food waste.

**Table 4:** Factors affecting food waste among college students (China, 2023, *n* = 407).

Variable	Level of food waste per meal		Frequency of food waste per week	
	OR (95% *CI*)	*p*	OR (95% *CI*)	*p*
Region of the University	0.944 (0.697, 1.279)	0.709	1.029 (0.754, 1.405)	0.857
Gender	0.842 (0.516, 1.373)	0.491	0.999 (0.605, 1.649)	0.997
BMI^#^	0.680 (0.497, 0.930)	0.016^*^	0.620 (0.443, 0.869)	0.005^*^
Grade	1.030 (0.834, 1.271)	0.784	1.085 (0.875, 1.346)	0.457
Scholarship	0.635 (0.336, 1.201)	0.162	0.582 (0.306, 1.110)	0.100
Hunger experience	0.847 (0.510, 1.407)	0.522	1.151 (0.680, 1.951)	0.600
Agricultural labor experience	1.008 (0.620, 1.638)	0.975	1.070 (0.647, 1.770)	0.793
Average monthly consumption (yuan)	1.438 (1.050, 1.968)	0.024^*^	1.216 (0.883, 1.676)	0.232
Attitude toward “Clean your plate campaign”	1.234 (0.773, 1.971)	0.379	1.298 (0.778, 2.163)	0.318
Household registration	1.693 (0.946, 3.032)	0.076	1.184 (0.656, 2.140)	0.575
An only child	1.416 (0.855, 2.347)	0.177	1.002 (0.597, 1.681)	0.995
Parental accompaniment	1.257 (0.567, 2.790)	0.573	1.014 (0.451, 2.281)	0.973
Family farming	1.035 (0.589, 1.821)	0.904	1.145 (0.638, 2.058)	0.650
Parental education	1.088 (0.872, 1.357)	0.454	0.973 (0.774, 1.223)	0.815
Dining place	1.062 (0.696, 1.622)	0.779	1.075 (0.693, 1.667)	0.748
Number of people eating together	0.962 (0.746, 1.242)	0.768	0.873 (0.672, 1.135)	0.310
Peers waste	1.924 (1.224, 3.022)	0.005^*^	1.617 (1.021, 2.561)	0.040^*^
Cafeteria vegetable quantity	1.287 (0.874, 1.895)	0.201	1.416 (0.935, 2.144)	0.101
Cafeteria meal quantity	1.348 (0.917, 1.981)	0.128	0.979 (0.654, 1.466)	0.919

^#^BMI, body mass index. According to the Chinese Public Health Standards, as follows: underweight, BMI < 18.5 kg/m^2^; normal weight, 18.5 ≤ BMI < 24.0 kg/m^2^; overweight, 24 ≤ BMI < 28.0 kg/m^2^; and obese, BMI ≥ 28.0 kg/m^2^. The results were shown as OR (95% CI). Statistical analysis was conducted using the Logistics multivariate regression analysis. ^*^p < 0.05 compared to the control group.

### 3.4 Effectiveness of labor education

We compared the changes in dining attitudes and behaviors of males and females after participating in labor education, and the results are shown in Table 5. Before labor education, there was no difference between male and female perceptions of cafeteria waste, but after the practice of labor education, female students were more likely to believe that cafeteria waste was still more serious (*Z* = −2.050, *p* = 0.040, Table 5, Q1). On the other hand, before and after labor education, there was a difference in food wastage among both males and females, both of which showed more serious wastage among female students. However, the improvement of students’ wasteful behavior was not obvious after labor education (Table 5, Q2 and Q3).

**Table 5:** Evaluation of the implementation effect of labor education for college students (China, 2023, *n* = 27).

Question		Before		After		Before vs. After
		Male	Female	Male	Female	
	*n*	12	15	12	15	–
Q1 Do you feel there is a lot of waste in the cafeteria?	Not serious	2	1	4	1	–
	A little serious, but acceptable	6	12	7	9	–
	More serious and requires measures	4	2	1	5	–
	It does not matter	0	0	0	0	–
	*Z*	−0.528		−2.050		−0.423
	*P*	0.598		0.040^*^		0.672
Q2 What is your level of food waste per meal?	Almost no waste	9	2	7	2	–
	Waste a little (<10%)	1	8	3	8	–
	Less waste (10–30%)	2	3	2	4	–
	Comparatively large amount of waste (30–50%)	0	2	0	1	–
	Large amount of waste (>50%)	0	0	0	0	
	*Z*	−2.690		−2.126		−0.303
	*P*	0.007^*^		0.034^*^		0.762
Q3 How often do you waste food each week?	0–1 time per week	11	4	11	5	–
	2–3 times per week	1	5	1	6	–
	4–5 times per week	0	4	0	3	–
	More than 5 times per week	0	2	0	1	–
	*Z*	−3.324		−3.023		−0.456
	*P*	0.001^*^		0.003^*^		0.648
Q4 Do you feel self-conscious about the waste caused by leftovers?	Regularly	7	3	8	7	–
	Occasionally	5	10	4	7	–
	Not usually	0	2	0	1	–
	Not at all	0	0	0	0	–
	*Z*	−2.185		−1.118		−1.363
	*P*	0.029^*^		0.264		0.173
Q5 What is your attitude toward your classmates wasting food?	Stop it now	1	2	4	4	–
	I do not like it, but I’m too embarrassed to stop it	4	3	6	5	–
	It does not matter. It’s normal	2	3	0	0	–
	It’s someone else’s business	5	4	0	3	–
	I’ll tell the student privately	0	3	2	3	–
	*χ* ^2^	4.621		4.117		13.831
	*P*	0.328		0.249		0.008^*^
Q6 Do you think the signs posted on the cafeteria about saving food work for you?	It does not work at all	2	3	0	0	–
	It works a little bit	2	4	1	6	–
	Sometimes it works	4	7	5	2	–
	Serve a valuable purpose	4	1	6	7	–
	*Z*	−1.177		−0.897		−2.274
	*P*	0.239		0.370		0.023^*^

The results were shown as frequency. Statistical analysis was conducted using the Mann–Whitney U test. ^*^p < 0.05 compared to the control group.

Before labor education, males and females had different attitudes toward food waste, and females were more likely to show indifference (*Z* = −2.185, *p* = 0.029, Table 5, Q4), and the difference in attitudes between males and females was not significant and did not improve after labor education. After labor education, students were less likely to be indifferent to their peers’ waste (*χ*^2^ = 13.831, *p* = 0.008, Table 5, Q5). Most of the students thought that the cafeteria slogans were still useful in warning about food waste. After labor education, the proportion of students who thought that the food-saving slogans posted in the cafeteria had “no effect at all” decreased from 18.52% (5/27) to 0, and the proportion of those who thought that they had “some effect” increased (81.48% vs. 100%, Table 5, Q6). The percentage of those who thought that “it is very useful” increased to 48.15% (13/27), and in general, after labor education, students thought that posting food-saving slogans in the cafeteria had “some good effects” (*Z* = −2.274, *p* = 0.023, Table 5, Q6).

In conclusion, after participating in the practical activities of labor education, the students’ views and practices toward their peers’ food waste have improved.

## 4 Discussion

Food is an essential human requirement. Food waste is a significant global problem. The majority of average consumers consider food waste to be a problem of social focus rather than as an environmental or economic problem (9). Environmental damage is not only caused by production but also by food waste since part of this waste is related to various environmental aspects such as the emission of greenhouse gases (10, 11), in turn, the prevention of food waste depends on consumer behavior. Therefore, food waste is presently gaining extra attention due to wastage itself leads to serious economic, environmental, moral, and social consequences (12). According to the United Nations International Children’s Emergency Fund (UNICEF) 2011 report, approximately 21,000 people perish daily due to hunger-related problem. On the other hand, nearly one third of all food produced is sent to landfills (13). Food waste has been identified as one of the major factors that constitute numerous anthropogenic activities, especially in developing countries (14). Reducing food waste is an important element of a sustainable environment and is the responsibility of every country and every individual.

For college students, their primary activities occur at school, and school cafeterias worldwide offer an opportune microcosm in which to educate on food and nutrition knowledge and change related behavior (15). Nevertheless, young adult college and university students, who have proven to be more wasteful (16). Therefore, this study focuses on observing cafeteria food waste among college students. In order to increase the representativeness, we selected college students in three regions to conduct a food waste survey. The results showed that food waste of college students varies in different regions, and East China is more serious. It may be related to the fact that the economy of this region is more developed, and college students generally have better family conditions, and they have a weak sense of thrift and frugality since childhood, and have not developed the habit of saving food. In addition, this part of the students’ purchasing and choice of food is on the high side, and these may lead to a greater tendency to food waste. This is similar to previous studies that found people with higher income families waste more than households with a lower income (17, 18).

Experimental evidence suggests that age and sex are important factors for food consumption and hence the generation of food waste (19). Our study found that compared with the lower grades students, the upper grades students have a higher level of food waste, considering that it may be because the lower grades students have a lower level of consumption and have just been separated from their families for a short of time, and they still show more rustic living habits. On the other hand, there is still a certain degree of freshness to the cafeteria, therefore, there is no obvious food waste shown. Correspondingly, the majority of the upper grades students have a higher level of living expenses, and those who have a higher level of living expenses tend to consume snacks with a higher frequency. We found that compared with male, female’s food waste is more serious, which may be related to the fact that females have to maintain a pretty figure, the previous study showed that females tend to improve their appearance image with the help of dietary adjustment (20), and individuals with appearance anxiety are more likely to suffer from anorexia nervosa and thus tend to waste food (21). On the other hand, females consume more snacks, which leads to a certain degree of decline in the amount of meals to the cafeteria, and the vast majority of students do not have the habit of packing leftover meals. However, it was observed that although female waste more than male (22), they are more predisposed to reduce food waste when compared to the latter (23).

Previous studies have suggested that food waste is shaped by multiple individual and social environmental factors (24, 25). Niaki et al. (24) has suggested that around 78% of respondents believed that students engaging more in socializing than in focusing on eating was the reason for food waste on the school campus (24). On the other hand, the most common reasons for throwing away food were exceeding the expiration data, storing food for too long, not having enough time to use up stored ingredients, and preparing too-large portions of meals (26, 27). Research has generally concluded that both low and high BMI can have an impact on emotion (28), this in turn affects the choice of food type and quantity.

This study suggested that food waste occurs less frequently among heavier college students. That is to say, fat college students can only consume large amounts to satisfy their normal metabolism, and thus are not prone to food waste. As the largest developing country in the world, China has seen a remarkable change in its social environment, which may influence the eating behaviors of college students. Compared to children and adolescents, college students may have greater access to their own money and much more freedom to make decisions about their eating (29). This study showed that the higher the monthly consumption, the more serious the food waste among college students. This is also an indirect indication that college students with better economic status are indifferent to food conservation. Recent research also suggests that other environmental factors, such as interpersonal relationships (30), might shape individuals’ food waste behavior. The results implied that college students are more likely to have food waste if their peers food waste. Because this peer effect is twofold. That is why group educational interventions are necessary for college students.

Wasted food, an indicator of systemic food system issues, is pervasive and must be addressed holistically. Educational programming, is one important tool among others for decreasing food waste and improving the footprint and overall justice of the global food system (31), among many other things, is an essential aspect of improving food-related behaviors (15). Educational institutions worldwide have recognized their ability to encourage food waste reduction and improve their own measures of institutional sustainability through campus food diversion programming (32). In the context of the new era, universities shoulder the important responsibility of cultivating talents for the country, and it is even more necessary to deploy labor education as an important task. The value of labor education is fully realized through students’ personal participation in labor practice. This study combined college students’ food waste and its influencing factors to carry out labor education, and the results show that after the labor education, college students’ attitudes toward their peers’ wastefulness have changed, and they resolutely resist this food waste situation. But we know that it takes time to change any behavior or habit. That’s why we are considering labor education on a larger scale and for a longer period of time.

Food waste is a significant global problem and young people need to be activated to change their behavior. This study can guide educational efforts and contribute to further research in this area. Therefore, education among students regarding food waste and its consequences should be considered (15, 33, 34). Of course, reducing food waste is a complex and long-term process and requires a shared responsibility between the state, society and even the family. In order to promote sustainable social development, China enacted “The Law of the People’s Republic of China Against Food Waste” in 2021, implying that the public will face penalties for food waste behavior. Therefore, based on the results of this research, government and college should constantly expand high-quality educational resources, it is possible to root the awareness of caring for the environment and saving resources in students’ instincts, and thus reduce food waste behavior. Furthermore, the government should launch a campaign on the theme of food conservation every year, so that awareness of environmental protection can cover all groups, and further guide the general public to develop the concept of honor in conservation and the habit of diligence and thrift among individuals.

However, this study also has several limitations. First, bigger sample sizes could give us a better insight into the student population. Furthermore, the sample will be more representative by adopting whole cluster random sampling. In addition, the number of students who participated in the labor education practice was small and the time was too short to clearly observe the change of labor education on college students’ food waste behavior in a short term.

## 5 Conclusion

Studying the phenomenon of food waste in colleges is conducive to the formulation of public policies to regulate the behavior of college students, and labor education is conducive to the establishment of a correct outlook on labor and a sense of conservation among college students.

## Data availability statement

The original contributions presented in the study are included in the article/supplementary material, further inquiries can be directed to the corresponding author.

## Author contributions

DW: Methodology, Writing – original draft, Writing – review & editing. KZ: Investigation, Writing – review & editing. XL: Investigation, Writing – review & editing. LX: Investigation, Writing – review & editing. ZY: Investigation, Writing – review & editing. PL: Funding acquisition, Supervision, Writing – review & editing.
